# A variational approach to niche construction

**DOI:** 10.1098/rsif.2017.0685

**Published:** 2018-04-11

**Authors:** Axel Constant, Maxwell J. D. Ramstead, Samuel P. L. Veissière, John O. Campbell, Karl J. Friston

**Affiliations:** 1 Laboratory of Experimental Psychology, Brain and Cognition Unit, KU Leuven, 3000 Leuven, Belgium; 2 Institute of Philosophy, KU Leuven, 3000 Leuven, Belgium; 3 Amsterdam Brain and Cognition Center, University of Amsterdam, Science Park 904, 1098 XH Amsterdam, The Netherlands; 4 Department of Philosophy, McGill University, 855 Sherbrooke Street West, H3A 2T7, Montreal, QC, Canada; 5 Division of Social and Transcultural Psychiatry, Department of Psychiatry, McGill University, 1033 Pine Avenue, Montreal, QC, Canada; 6 Department of Anthropology, McGill University, 855 Sherbrooke Street West, H3A 2T7, Montreal, QC, Canada; 7 Independent Researcher, Victoria, BC, Canada; 8 Wellcome Trust Centre for Neuroimaging, University College London, 12 Queen Square, London, WC1N 3BG, UK

**Keywords:** evolutionary biology, niche construction theory, ecological inheritance, variational free-energy principle, active inference, situated learning

## Abstract

In evolutionary biology, niche construction is sometimes described as a genuine evolutionary process whereby organisms, through their activities and regulatory mechanisms, modify their environment such as to steer their own evolutionary trajectory, and that of other species. There is ongoing debate, however, on the extent to which niche construction ought to be considered a bona fide evolutionary force, on a par with natural selection. Recent formulations of the variational free-energy principle as applied to the life sciences describe the properties of living systems, and their selection in evolution, in terms of variational inference. We argue that niche construction can be described using a variational approach. We propose new arguments to support the niche construction perspective, and to extend the variational approach to niche construction to current perspectives in various scientific fields.

## 1. Introduction

Niche construction refers to any (implicit or explicit) modification by organisms of the (biotic or abiotic) states of the niche that they and others inhabit [1–3]. Niche construction theory (NCT) casts niche construction and ecological inheritance (the inheritance of selection pressures modified by organisms) as bona fide evolutionary processes acting in tandem with standard evolutionary processes like natural selection. NCT studies the way organisms (i) generate non-random biases on selection pressures, (ii) stabilize environmental conditions [1], (iii) and secure organism–niche complementarity (adaptation) [2]. Central to NCT is the view of reciprocal causation, which refers to the fact that developing systems can be both the products and causes of evolution [4].

Reciprocal causal dynamics that pertain to niche construction are expressed at two temporal scales: the timescale of ontogeny and the timescale of phylogeny [5]. Developmental niche construction (DNC) allows for the optimization of phenotypes over ontogenetic timescales, via feedback interactions between organisms and resources of the niche (e.g. parental care and culture acquisition). Selective niche construction (SNC) acts in tandem with natural selection to optimize phenotypes over intergenerational timescales by modifying selection pressures. It concerns consequences of feedback interactions that span over multiple generations (e.g. ecological inheritance).

There is much debate among proponents of the modern synthesis (MS) in biology as to whether niche construction is a bona fide evolutionary *process* [6,7]. Proponents of MS acknowledge the *effects* of constructed niches on evolutionary processes [8], but tend to underemphasize their significance. For instance, environmental modifications emanating from organisms' activity are generally cast as being on a par with random environmental changes, e.g. the effects of tsunamis, or volcanic eruptions [8].

The aim of this paper is to review and integrate recent theoretical work on niche construction in neuroscience, biology and anthropology [1,5,9–18]. We present the theoretical foundations and modelling heuristics for a novel computational framework for niche construction dynamics. The variational approach casts evolutionary processes in general as approximate Bayesian (variational) inference, serving the attainment of maximum attunement between the states of an organism and the states of its environment [19] (i.e. the occupation of local free-energy minima in the organism–niche state space). For instance, natural selection can be cast as a process of variational inference, in the sense that the fittest phenotype for a given ecological niche is also the one most likely to be found in that niche and populate it, given a set of environmental constraints. Somatic changes can be cast as a Bayesian update of the parameters of the model embodied by the organism, and genotypic change through natural selection can be cast as a process of Bayesian model selection [20–24] (see the electronic supplementary material for further details).

Central to this paper, the variational (free-energy) approach supports the view of niche construction as a bona fide evolutionary process. Specifically, it may provide a principled method of quantifying the complementarity that obtains between organisms and their niche via niche construction, as well as a computationally tractable definition of algorithmic information [25] and its transmission via ecological inheritance [3]. We discuss the implications of this framework with respect to extensions of the niche construction perspective in developmental psychology (e.g. [26]). Overall, the variational approach could provide a promising modelling tool for research on the niche construction perspective.

## 2. The scope of niche construction theory

In this section, we review recent developments in the literature on NCT, namely the views on DNC and SNC, and present the standard critiques of NCT, which we interpret under the lens of the variational approach in the final section of the paper.

### 2.1. Selective niche construction

From the point of view of SNC, the ecological niche comprises the set of environmental factors that causally influence the inclusive fitness (rates of survival and reproduction) of organisms, emphasizing those produced by organisms. SNC encompasses all such environmental changes, ranging from the constitution of a layer of warm, moist air around homeothermic organisms [27], or the ability of earthworms and benthic diatoms to self-impose selection pressure through changes in structure and chemical composition of their local environment [28,29], to development of complex behavioural patterns like human communication and cultural systems [30–33].

It is important to note, however, that while any modification to the environment is part of niche construction, only those that have an impact on the scales of development, ecology or evolution ought to be considered as meaningful for NCT. For instance, one might argue that to induce a layer of moist air is not a process powerful enough to have a significant impact on the relevant scales. Advocates of NCT generally argue that the extent to which one should cast a given kind of modification of the environment as relevant niche construction is ultimately an empirical matter.

### 2.2. Developmental niche construction

The developmental niche, as a temporal subset of the selective niche, comprises the set of environmental parameters that enable the development of organisms over ontogeny [5]. It is a reliably inherited, intricate structure of physical, social and epistemic resources that enables the reconstruction of the species-typical developmental trajectory (i.e. the adaptive life cycle), as well as the production of adaptive phenotypic variations [34]. By providing reliably inheritable and contextually flexible inputs for developmental plasticity, for instance by canalizing certain forms of phenotypic accommodation, DNC scaffolds phenotypes at mechanistic and ontogenetic scales [5].

Exogenetic factors may comprise complex behavioural patterns like parental care, stimulation of offspring and social learning, and function as crucial resources for normal development ([5,31,35–37], cf. the earlier concept of ‘ontogenetic niche’ [38]). Other exogenetic resources in the developmental niche include myriad social and transgenerational relationships, e.g. cooperative breeding [39], shared patterned cultural practices [40,41] and regimes of attention [42]. Crucially, these factors enable organisms to acquire the expectations (and, in humans, the norms) that regulate communities, and to learn the practices that have adapted to the particular niche through cultural evolution and culture–gene coevolution [37].

### 2.3. Standard critiques of NCT

Proponents of the MS are critical of NCT. Part of their motivation is that they understand causation in evolution as unidirectional. On this account, evolutionary processes begin with environmental (biotic and abiotic) selection pressures, and culminate in genotypic changes that secure adapted phenotypes. Although MS acknowledges some cases of reciprocal causation in evolution (e.g. sexual selection), it casts environmental modifications stemming from organismic activity as being the same in kind as those that stem from random natural environmental changes, i.e. they do not count as evolutionary processes properly speaking [6,7].

With standard evolutionary processes (e.g. natural selection), traits that enhance fitness are systematically retained, and those that do not are lost. This explains the appearance of adaptive design. With niche construction, it is unclear whether or not niches adapt the environment to the organism in a systematic way, because constructed environments can lead both to increase and decrease of reproductive success [6]. Moreover, the standard model of evolution allows scientists to make predictions about the effects of natural selection on design, and on the optimization of inclusive fitness [43]. Because of the lack of systematicity of niche construction effects, NCT might not be able to generate predictions about adaptation.

Perhaps the most extensive critique of NCT was offered by Richard Dawkins ([44], cf. [45]). He dismisses NCT's claim of cyclical causality between the genome and environmental modifications induced by organisms. On his account, most of the effects of organismic activity of the environment are just ‘too loose and vague to count as … true niche construction’ [44]. For instance, beaver kits (and grandkits) may gain reproductive success from a well-constructed dam, and this might encourage the preservation of alleles underlying the behaviour of dam construction. Effects on other species, however, are not directly relevant to the beaver's, and should, therefore, be considered as mere by-products. One ought not to confusedly cast the dam as an adaptation and the dam as a by-product, or confuse ‘beaver dams with the beaver's dungs’ [46]. The scope of NCT, however, goes beyond the adaptations\by-products dichotomy to emphasize the underestimated role of the latter in evolutionary feedbacks.

The variational approach explored in the remainder of this paper puts into perspective the relevance of the distinction between adaptations and by-products. Consequences of an organism's activity, in either case, can be framed as contributing to the attainment of maximum statistical attunement with the environment, which we cast below as the minimization of variational free energy.

## 3. Variational free energy and the dynamics of life

In this section, we present the conceptual foundations and motivation for the variational (free-energy) principle in relation to biological self-organization (see §4.1. for a formal description). The main ideas discussed here are the statistical conception of the phenotype, and the dynamics underwriting its organization.

### 3.1. A statistical conception of the phenotype

The variational free-energy principle (FEP) has been applied fruitfully to yield a statistical conception of the dynamics of life [47]. The key idea behind this principle is that the bounded set of *characteristic* states in which an organism maintains itself most of the time, and in which it is *most likely* to find itself, can be interpreted as the set of its *phenotypic states*, i.e. its specific repertoire of functional or adaptive states, its physiology and morphology, and its sensorimotor patterns. Since these characteristic states are visited more frequently than others, they are associated with a higher probability than other states (e.g. a fish is more likely to find itself in water than anywhere else). This statistical conception of the phenotype is supported by simulation studies of the emergence of biological self-organization and morphogenesis [47,48], and further evidence comes from the study of early myelopoiesis in real biological systems (e.g. [49]).

Phenotypic states subsume all states of an agent, from quickly fluctuating ones (e.g. its temperature or visually evoked responses), to slowly fluctuating states that constrain fast dynamics (e.g. its morphology, structure and neuronal connectivity). Later, we will consider this distinction in terms of the distinction between stable and unstable dynamics in synergetics [50], where the latter (unstable, slow) modes correspond to *order parameters*, and will be associated with ‘traits’ of phenotypes, which can include states of the niche. Thus, the conception of the phenotype that interests us here can be framed in terms of a *joint phenotypic space* that includes states internal (e.g. phenotypic states) and external (e.g. phenotypic traits) to the organism, biotic and abiotic.

### 3.2. Motivation and problem

Organisms are open systems. Yet, they maintain their organization over time in the face of environmental perturbations. Therefore, one must assume (*a priori*) that they manage to limit the entropy of their states [51]. Technically, entropy (as an information theoretic quantity) is the long-term average of self-information or ‘surprisal’. Surprisal reflects the likelihood of an outcome—it is the negative log probability of any sensory state being encountered. Organisms that maintain their continued existence spend most of their time in attracting states, and will, therefore, have a low average surprisal and low entropy. Note that one could also define entropy (as a thermodynamic quantity) in the following way. Under ergodic assumptions (that is, under the assumption that the system has properties that can be measured more than once), surprisal is equivalent to a thermodynamic potential energy [52]. Although the stationary distributions involved do not necessarily have a Boltzmann–Gibbs form, recent developments finesse the difficulties in identifying potential functions that play the role of an adaptive landscape: see [53] for a technical discussion. Only the information theoretic definition concerns us directly in this paper.

**Figure 1.**
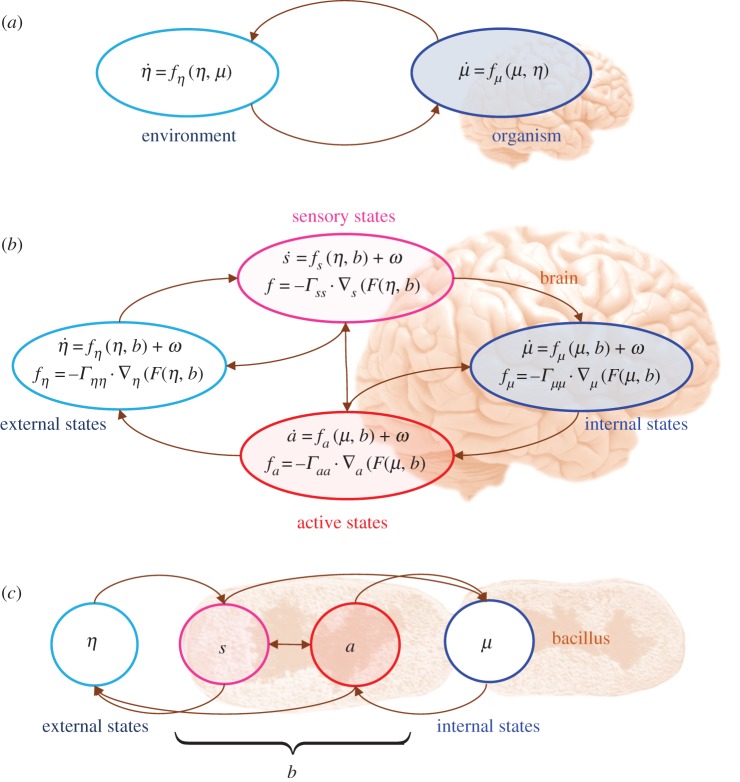
Markov blankets. These schematics illustrate the partition of states into internal and hidden or external states that are separated by a Markov blanket (*b*) comprising sensory and active states. (*a*) A schematic that captures the relations of reciprocal causation between the organism and its environmental niche [54]. Changes in states of the organism over time are a function of the immediate state of the organism (*μ*) and its environment (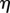), and, reciprocally, changes in the states of the organism's environmental niche over time are a function of the current state of the environment and the organism. The form of this dynamical coupling can now be specified by appeal to the FEP. This introduces a separation of organismic and environmental states (i.e. internal and external states) via an intervening Markov blanket of active (*a*) and sensory states (*s*). Once the Markov blanket is in place, the non-equilibrium steady-state dynamics are prescribed by the Fokker Planck equation that can be expressed in terms of gradient flows on variational free energy. (*b*) This partition (and flows) as it would be applied to action and perception in the brain, where active and internal states minimize a free-energy functional of internal and blanket states. The ensuing self-organization of internal states then corresponds to perception, while action couples brain states back to external states. However, because of the antisymmetry of the conditional dependencies implicit in the Markov blanket, we can also express external dynamics as a gradient flow of a free-energy functional of external and blanket states. (*c*) Shows exactly the same dependencies but rearranged so that the internal states are associated with the intracellular states of a cell, while the sensory states become the surface states of the cell membrane overlying active states (e.g. the actin filaments of the cytoskeleton). For simplicity, we have admitted the solenoidal flow in this figure. For a more complete account, see [47]. (Online version in colour.)

Because surprisal depends on hidden causes in the environment, the organism (e.g. its brain) cannot *directly* assess it. Here is where the variational free energy comes in. Mathematically, it constitutes an upper bound on surprisal, and thereby implicitly bounds its average, namely, entropy. Variational free-energy reports a deviation from, and is conditioned on, a set of priors, which are operationalized in a hierarchical, probabilistic *generative model* of the hidden causes of sensory inputs (observations) [20]. Priors are probability distributions or Bayesian beliefs that provide constraints on lower levels, in hierarchical models. Because they are themselves parametrized, they can be optimized via hyperparameters that depend upon the sensory observations (refer figure 1 and §4.1 below for technical details).

A generative model is a mapping from sensory observations, action policies, to external (hidden) causes. These causes constitute the *generative process*, which describes the transitions among hidden causes in the world (including the organism's own actions) that generate sensory inputs [55]. The generative model is conditioned on the prior (Bayesian) beliefs of the organism. These beliefs are parametrized by a density that is encoded by the internal states of the organism (e.g. brain states). See figure 1 for a discussion. This density represents the ‘best guess’ of the organism as to the causes of its sensations. Updates of the generative model conform to the principles of variational inference. This involves approximating the generative process by optimizing parameters of the internal density, with respect to the free energy bound on Bayesian model evidence (sometimes referred to as an evidence bound). This bound means that if one changes some probability distribution (i.e. beliefs) over the causes of observed data to minimize variational free energy, one is effectively maximizing model evidence [52].

Thus, although it cannot track surprisal directly, the organism can track a proxy quantity (an upper bound), the variational free energy. By minimizing free energy (or maximizing model evidence), the organism can estimate surprise, and thereby avoid deleterious phase transitions and maintain its phenotypic organization. Hence, the long-term, evolutionary (distal) imperative for the organism to minimize entropy translates into the short-term (proximal) imperative to avoid surprisal by minimizing free energy (see §4.2. for the mathematical details).

### 3.3. Maximizing model evidence through action and perception

Active inference is the self-evidencing process by which the organism garners and produces evidence for the generative model that it instantiates by existing. At the scale of organisms interacting with their niche, active inference is realized in patterns of action and perception. Action allows the organism to gather more precise (Bayesian) model evidence: sensory samples reduce uncertainty with respect to the causes of sensory states. In turn, the role of perception, sometimes called ‘perceptual inference’, is to update the system's priors while informing action. This process amounts to minimizing the surprisal *expected following an action*, where expected surprisal is also known as uncertainty. In other words, all action is—at one level—in the game of resolving uncertainty and minimizing surprisal. Given the statistical conception of the phenotype described above, the upshot is that we can interpret active inference as the generalized homeostasis and allostasis of an organism [21,56].

Relating these ideas to the core of our discussion, variational free energy is minimized at all scales of self-organization; from the ensemble behaviour of macromolecules to evolutionary dynamics [57]. It is a purely information theoretic construct that generalizes thermodynamic free energy. It can provide an upper bound on the (log) evidence for the exchange of a structure like a cell with its environment. This evidence can either be interpreted as self-information (a.k.a. surprisal), such that the time integral of self-information becomes the entropy of environmental exchange. By interpreting the evidence in relation to a generative model, one can associate the biophysical states of any living system (cell, brain, phenotype, species, etc.) with the sufficient statistics of the generative model.

The variational free energy is then a functional of the environmental (e.g. sensory) input to the system and the probability distribution over causes of that input parametrized by the system's internal states (e.g. intracellular concentrations, neuronal connectivity, anatomical infrastructure, etc.). Crucially, the generative process generating the input may be formally distinct from the generative model of that process. This construction means that the same free-energy functional is minimized at different spatial and temporal scales, depending on how the system is defined. This minimization describes, for example, the chemotactic behaviour of *Escherichia coli*, learning in our brains, institutional organization, etc.: see [47,52] for a detailed discussion and [48] for a worked example in morphogenesis.

From the perspective of the organism, minimizing free energy through active inference may feel like constructing ‘designed’ environments [58]. From the niche's perspective encoding the traces of organismic activity, it is just ‘learning’ about the phenotypes that inhabit it: both niche and phenotype are self-evidencing. Mathematically, action on the environment is the same as the niche sensing an organism. This is key to our treatment in what follows. If both the niche and organism are ergodic, then they must both conform to the free-energy principle. This implicit symmetry or circular causality means that free-energy bounding dynamics are also a special case of generalized synchrony, e.g. two swinging pendulums attached to a beam that synchronize over time, or between a phenotype (e.g. me) and (e.g. my) niche, which is usually constituted by other sentient systems (e.g. like me). This synchrony is what we associate with (developmental) niche construction dynamics generating ecological inheritance.

## 4. A variational approach to niche construction

This section summarizes the conceptual and mathematical analysis that underlies the variational principles supporting the modelling heuristics for the variational framework of niche construction.

### 4.1. Niche construction, variational free energy and generalized synchrony

This section summarizes the conceptual and mathematical analysis that underlies the variational principles we are pursuing. The FEP furnishes an interpretation of an agent's internal dynamics as a gradient flow on a free-energy functional of sensory states and an approximate posterior based on a generative model of external dynamics (and how they map to sensory states). This means that the internal states of an agent acquire a Bayesian mechanics via an implicit (generative) model of its environment. This interpretation rests on a distinction between states that are external and internal to a given agent. This (statistical) separation requires the existence of something called a *Markov blanket*—that itself can be divided into active and sensory states [47,59,60]. The only requirement (that licenses a distinction between internal and external states) is that internal states cannot directly influence sensory states—and external states cannot directly influence active states (figure 1).

The key move now is to appreciate that the same Bayesian interpretation can be applied to the external states. This follows from the antisymmetry of the conditional dependencies that define the Markov blanket. In other words, we can relabel internal states as external states and relabel sensory states as active states. In so doing, the conditional independencies remain unchanged (figure 1). This means that there must be a description of niche dynamics, where the environment models how the agent's internal dynamics are generating its active states. From the perspective of the environment, the agent's active states now become sensory states and sensory states become active states. The crucial observation here is that the agent and eco-niche share the same Markov blanket and, therefore, mathematically speaking, must be *inferring each other*.

On this view, one can now consider the ultimate (non-equilibrium) steady state of reciprocal exchange between the agent and its environment. We know that the dynamics of both external and internal states can be cast as gradient flows on the respective free energies of the agent and environment. So, under what conditions would their free energies be minimized? The following lemma suggests that this non-equilibrium steady state corresponds to a generalized synchrony between the agent and niche.

**Lemma 4.1. (generalized synchrony).**

If the internal and external states of a random dynamical system exhibit generalized synchrony, then their variational free energy is jointly minimized with respect to internal and external states.4.1
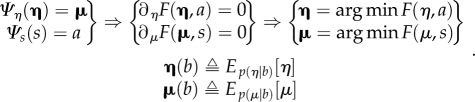

Here, 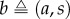 are *blanket* states that comprise *action* and *sensory* states, respectively, while the *external* and *internal* states are denoted by 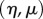. Generalized synchronization implies a synchronization manifold: 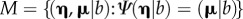, which has been conditioned on the blanket state. The existence of this (conditional) manifold is assured because for every given blanket state, there is an expected external and internal state. The equations above imply that the existence of a synchronization manifold renders the expected states minimizers of their respective variational free energies.

**Figure 2.**
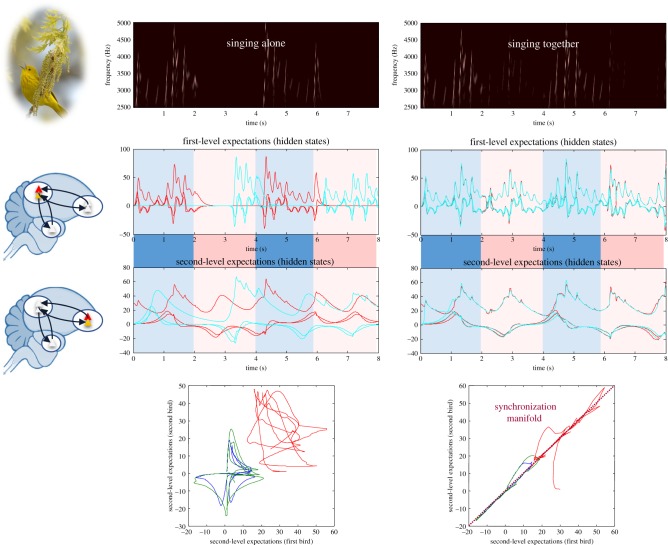
A duet for one. In this simulation of free-energy minimization, two birds with the same generative models—but different initial expectations—sing for 2 s and then listen for any response. In the right panels (*singing alone*), the birds cannot hear each other (because they are too far apart) and the successive epochs of songs diverge due to the sensitivity to initial conditions implicit in their (chaotic) generative models. The upper panels show the sonogram heard by the first bird. Because this bird can only hear itself, the sonogram reflects the proprioceptive predictions based upon posterior expectations in the HVC (*first-level expectations*) and area X (*second-level expectations*). These anatomical designations are based upon the hierarchical generative model illustrated with the insets (left). The posterior expectations for the first bird are shown in red as a function of time—and the equivalent expectations for the second bird are shown in blue. However, when the two birds can hear each other (*singing together*), the posterior expectations are encoded by internal states show identical synchrony at both the sensory and extrasensory levels—as shown in the middle panels. Note that the sonogram is now continuous over successive 2 s epochs, because the first bird can hear itself and the second bird. The ensuing synchronization manifold is shown in the lower panels. These plot the second-level (area X) expectations in the second bird against the equivalent expectations in the first. The left panel shows chaotic and uncoupled dynamics when the birds cannot hear each other, while the right panel shows the generalized (identical) synchrony that emerges when the birds exchange sensory signals. The different colours correspond to the three hidden states for each bird. The synchronization manifold for identical synchronization corresponds to the (broken) diagonal line. See [63] for details.

**Proof**

(heuristic): for simplicity, we will deal with the case of *identical synchronization*. Identical synchronization implies that there is an identity mapping between the external and internal states and their influences on each other [61,62]. This can be expressed as a synchronization in which the sensory states of the agent (i.e. active states of the environment) become the active states of the agent (i.e. the sensory states of the environment) and both are perfectly correlated. The synchronization manifold can be regarded as a surface that would be traced out if we plotted the states of the agent and environment against each other (figure 2 for example).

The free-energy principle says that the flow of internal can be expressed as a gradient flow on a free-energy functional of posterior beliefs about external states, 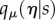 encoded by internal states, and *vice versa* for the external states:4.2
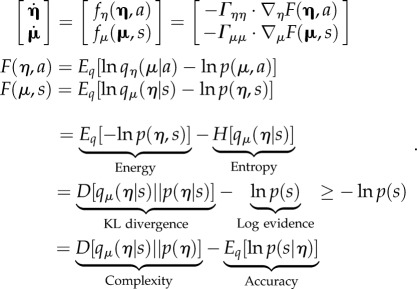

The free energy has been written here (for internal states) in several ways that provide a useful interpretation. The first decomposes the free energy into an energy and entropy term by analogy with statistical thermodynamics. The second rearrangement is more common in Bayesian statistics and machine learning. It shows that when the posterior beliefs are the true posterior density 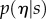, their KL divergence disappears and the free energy becomes negative log evidence 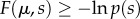, namely the negative log probability of sensory states, under the generative model of hidden causes and sensed consequences: 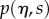. This is why the variational free energy is sometimes called an *evidence bound*—because it is always greater than negative log evidence [64,65]. The final rearrangement shows that minimizing free energy is effectively the same as finding accurate explanations that are minimally complex; i.e. that conform to Ockham's principle.

Under identical synchronization, 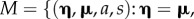 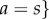 the gradient flows in equation (4.2) are identical everywhere. This implies that the underlying free-energy gradients are isomorphic on either side of the Markov blanket. In turn, this means that the generative model entertained by the agent about its environment is exactly the same as the generative model entertained by the environment about the agent. Furthermore, both agent and environment generate sensory and active states that are congruent with their shared generative model. This implies that the posterior beliefs (i.e. densities) must be veridical:4.3
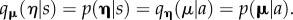

Substituting this into the expressions for free energy above shows that the divergence term disappears, and the free energy attains its lower bound.4.4
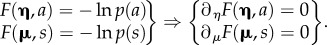

In other words, the generative model of both agent and niche are the veridical explanations of their respective sensory fluctuations and the variational free energies of both are jointly minimized with respect to the posterior beliefs parametrized by their respective states.

**Remark 4.1.**

For simplicity, we have just dealt with the situation of identical synchrony. Clearly, there will be formal asymmetries in the external structure of the environment and the internal structure of the agent. The above arguments must, therefore, be generalized (heuristically), such that the synchronization between internal and external states is generalized. Usually, in dynamical systems theory, generalized synchronization is considered in the light of skew product systems (i.e. master–slave systems) [61]. The twist here is that there is a bidirectional coupling that is actively maintained. This active maintenance rests upon a corollary of generalized synchrony; namely, that there is a generalized synchrony between the active and sensory states from the point of view of both the agent and niche. In other words, for generalized synchrony to emerge (i.e. for the minimization of joint free energy) it is necessary that both systems supply the right sort of sensory impressions that enable them to implicitly infer each other.

### 4.2. Minimal simulation of organism–niche synchrony

Clearly, the above construction starts to look like a model of communication. Indeed, the notion of generalized synchrony across a shared Markov blanket has been used previously to model communication in computational neuroscience—and resolve the problem of neuronal hermeneutics (i.e. inferring what was meant by reference to a shared narrative). Figure 2 illustrates the generalized (identical) synchrony that emerges when two synthetic songbirds can hear each other singing [63].

In the present setting, this can be regarded as a minimal simulation of the coupling between an agent and its niche, where the niche is another agent. Niche construction, in a broad sense, involves the modification of both biotic and abiotic ‘agents’. As discussed above, those modifications amount to learning via sensory and active (active inference) exchanges, also in a broad sense, among agents populating the niche.

This perspective on mutual influence—that is an emergent property of minimizing (joint) free energy—can be applied to multiple agents. A nice example of this is the joint free-energy minimization exhibited by simulations of cells that have a common (generative) model of their place within a tissue or organ; see [48,60] for details. In short, the same form of generalized synchrony (to a point attractor) can be used to model morphogenesis, where the environment is constituted by other agents with Markov blankets (i.e. other cells), who share the same generative model. This example is particularly interesting, because the evidence for the shared model now becomes the lower bound for the joint free energy [48].

The mathematical image of generalized synchrony, cast in terms of variational free-energy minimization, resonates nicely with earlier formulations of self-organization in cybernetics; namely, the good regulator theorem [66]. Here, the good regulator theorem applies not just to the regulator (agent) but also the (environmental) system that is being regulated (i.e. niche), conferring them both the status of agent. Clearly, to become a good model of one's niche, one needs to infer its causal structure.

In terms of the Bayesian mechanics above, this corresponds to a slow gradient flow on variational free energy averaged over time (in a path integral sense) [67,68]. In other words, there are certain states within and beyond the Markov blanket that constitute *order parameters* [50,69], or parameters of the generative model that are subject to the same free-energy minimizing pressures as fast fluctuations normally interpreted in terms of inference. The distinction between *phenotypic states* and *traits* maps to the distinction between the internal states that fluctuate quickly and slowly, where it is tempting to associate *phenotypic traits* with the order parameters of synergetics [50,70]—evolving over very slow (somatic or developmental) timescales. In relation to the statistical conception of the phenotype described earlier (cf. §3.1.), and the organism–niche synchrony described above, we can associate some of these phenotypic traits to order parameters that pertain to the material setting of the physical environment. These undergo even slower fluctuations caused, notably, by the niche construction.

**Figure 3.**
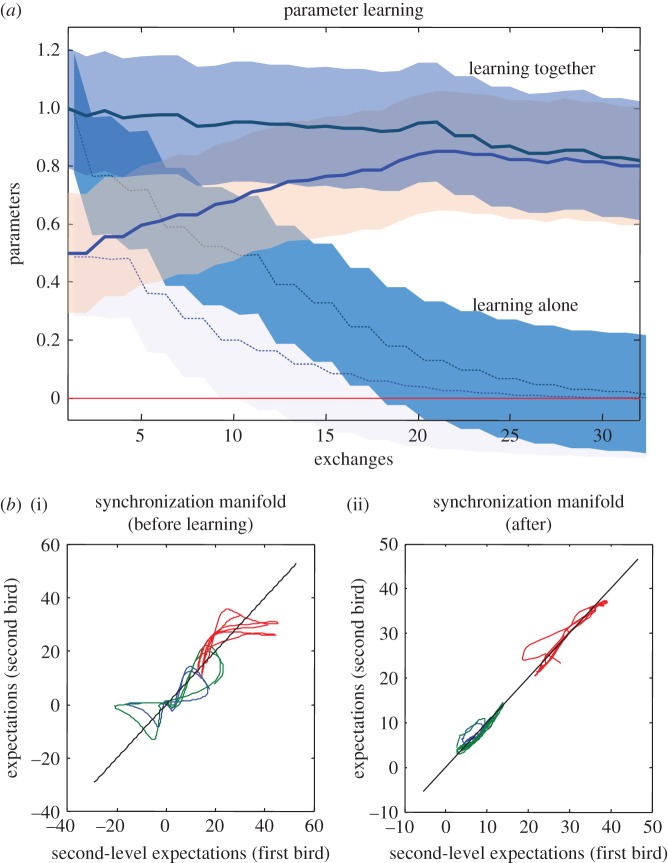
Learning and communication. (*a*) Shows epoch by epoch changes in the posterior expectations (lines) of an order parameter of the first bird (blue) and second bird (green) determining the (chaotic) structure of the songs of the sort shown in figure 2. The shaded areas correspond to 90% (prior) Bayesian confidence intervals. The broken lines (and intervals) report the results of the same simulation, but when the birds could not hear each other. (*b*) Shows the synchronization of extrasensory (higher) posterior expectations for the first (i) and subsequent (ii) exchanges, respectively. This synchronization is shown by plotting a mixture of expectations (and their temporal derivatives) from the second bird against the equivalent expectations of the first bird, where this mixture is optimized assuming a linear mapping. In this example of perceptual learning via free-energy minimization, the second (green) bird had more precise beliefs about its order parameter and, therefore, effectively, taught the first third. Note that in both scenarios, learning converges to the same value resulting in a generalized synchrony between self and non-self; i.e. beyond self-organization. See Friston & Frith [71] for details.

We introduce the distinction between *fast inference* and *slow learning* because simulations suggest that important asymmetries in the way that the agent learns about the environment—and the environment learns about the agent—can have profound effects on niche construction. In brief, if the (implicit) generative model of the environment holds very precise beliefs about certain dynamics, then the organism will come to learn the environment more quickly than the environment will learn about the agent. Conversely, when action upon the environment effectively teaches the environment about the agent—and the environment holds imprecise beliefs about the agent—the environment will yield to the agent's beliefs more readily (cf. results summarized in figure 3 [71]). The asymmetry in learning between organisms and their environment will be central to our discussion of ecological inheritance in §5.2. In brief, the asymmetry in learning rate between the environment and organisms allows material features of the environment to ‘retain’ regularities in behavioural patterns of groups of organisms unfolding over long timescales (e.g. cultural practices). These are ‘encoded’ through fast inference and niche construction by individual organisms. These may be viewed as order parameters encoded in the material features of the environmental, which are part of the joint phenotypic space (including slowly fluctuating traits, and quickly fluctuating states), and which are passed on through ecological inheritance.

In the present example of the two birds singing to each other, the (order) *parameters* of the two respective generative models were optimized with respect to their respective variational free energies. Crucially, one bird had relatively precise beliefs about the autonomous dynamics generating birdsong, while the other had less precise beliefs. The first bird, therefore, essentially learned from the second. On the current argument, we can associate the agent and environment with either bird, to introduce the notion that the environment may learn about the agent or the agent can learn about the environment, or that both can occur at the same time. The simulations of generalized synchrony (using birdsong) reported in this paper can be reproduced using the academic software available from http://www.fil.ion.ucl.ac.uk/spm/software/. Typing DEM at the MATLAB prompt will invoke a graphical user interface. The simulations can be reproduced by selecting *Birdsong duet* button. This allows users to examine the code (*DEM_demo_duet.m*) and subroutines—and customize them at their discretion.

Here, the ensemble that realizes the generalized synchrony consists in the entire population (the two birds) along with their local environment (for each bird, the other bird). In the simulation, the free energy of one bird is a functional of (sensory) signals from the environment (the other bird), while the free energy of the environment depends upon (action) signals from the bird. In principle, this equips the bird–environment system, or niche, with a measure of *complementarity* in relation to the phenotypes it hosts. Because the free energies of each bird and their respective environment (the other bird) are extensive quantities (i.e. they can be added together), we have, in principle, a way of scoring the coevolutionary complementarity among the various biotic and abiotic agents of a niche, which is simply the sum of their respective variational free energies. Increase in complementarity correlates with decrease in free energy. Crucially, this measure transcends any *particular context*, and can be compared quantitatively at different spatio-temporal scales (e.g. between two agents, two groups, two species).

This concludes our formal analysis. For further details, see [47] and [72] for a complementary treatment of self-organized criticality (i.e. critical slowing), variational free-energy minimization and generalized synchrony. We now turn the key role of *precision estimation* in determining the level of symmetry of exchange between agents and environment.

### 4.3. Niche construction and synchrony: a mechanism for meta-learning

The FEP understands precision estimation (the estimation of the reliability of sensory information) as a meta-learning mechanism, because it allows the organism to know what worth learning [73]. Learning precision is a crucial part of the attunement to causal regularities in the environment. For instance, humans have evolved a host of phenotypic expressions, from highly visible white sclera and gaze tracking abilities, to cultural ‘prestige cues’, which optimize the process of learning from whom and what to learn [37].

Constructed aspects of the niche can play an important role in learning, and estimating precision. Consider ‘desire paths’ in humans [74]. Such paths can be the by-products of people cutting through the grass rather than following the paved road. Over time, a dirt trail might form, thereby attracting additional pedestrians and reinforcing the trail in a looping manner, which will further steer future interactions between the users and their niche.

In this example, niche construction is mostly implicit: pedestrians need not be aware that they are constructing a path, or aware of how their action on the environment can benefit them, or impact others. They merely act in accordance both with local constraints, and with respect to their expectations (e.g. the desire to reach the extremity of the park). This highlights the fact that in general, we can expect physical changes produced through active inference to be consistent with, or complement the organisms' expectations.

Lasting physical changes to the environment produced via niche construction function as high fidelity, precise sources of information. By actively engaging their environment, organisms—and indeed, entire populations—fit their niches to their prior expectations, e.g. in desire paths. Information relevant to the generation of adaptive responses is encoded in the physical environment (cf. [18]), thereby directing the learning of causal regularities, and aiding the organism in managing uncertainty (cf. [75–78]) (e.g. there is a high probability that I end up faster to the other extremity of the park if I follow this trail). Salient aspects of the niche modulate the attention of organisms and direct active inference [42,79,80] by grounding learning in the socio-material fabric: constructed aspects of the niche function as meta-learning mechanisms and encode *salience*.

We use ‘salience’ here in a very particular and formal way that is consistent with the active inference scheme. Salience is the information gain, reduction of uncertainty, Bayesian surprise, or epistemic value that constitutes an important part of expected free energy. In brief, certain environmental cues (e.g. signs and semiotics) attain salience or epistemic affordance because they indicate the actions that will minimize expected free energy or, more simply, resolve uncertainty (see [81] for details). This facilitates substantially the modelling of the environment by the organism.

An important function of environment-based meta-learning is that it enables organisms to reduce cognitive demands, and in a sense ‘upload’ the computational burden of salience estimation to the environment itself. Hence, it is no stretch to say that learning what is salient or has epistemic affordance does not rest solely upon the learning of brain, or body-based priors, but also on carving out a specifically constructed niche through the traces left by reiterated action (of self and others, past and present). This could be an implicit strategy by which organisms save on metabolic resources. In the parlance of free-energy minimization, uploading information into the niche translates to minimizing complexity and associated thermodynamic costs of computation via the Jarzynski inequality [82,83]. Heuristically, it eases the decision-making process by reducing uncertainty as well as the need for metabolically expensive, internal (neural) information processing. Uploading also enables the reduction of model complexity over time by constraining the hypothesis space that the organism has to model, narrowing the range of possible priors, and thereby increasing thermodynamic efficiency [84].

## 5. Applications and predictions

In this section, we discuss the variational approach to niche construction in relation to situated learning in developmental psychology and ecological inheritance. We revisit the standard critiques of NCT under the lens of the variational approach.

### 5.1. Variational niche construction and situated learning

The idea of the niche as supporting learning is consonant with new perspectives on niche construction in developmental psychology, especially with the situated learning paradigm (e.g. [26]). As Flynn and colleagues point out, ‘the niche into which we are born … in part, dictates what we learn’ [26]. One of the functions of the developmental niche is to support situated learning, defined as the process by which an organism comes to acquire the knowledge necessary to integrate a ‘community of practice’ [85].

This requires of organisms that they engage conventionalized patterns of collective activity, grounded in material artefacts and other physical aspects of the niche, which have been transmitted to allow for the progressive integration of the organism as a legitimate member of the community [42,85]. From the point of view of NCT, the engagement of the constructed environment inherited from the community of practice enables situated learning, and influences the selection of norms, habits, and values modulating attention ([26,41], cf. the concept of regime of attention [79]).

By providing opportunities for learning how to learn, the developmental niche does not merely indicate what information ought to be learned, but also how best to learn it [26]. As a meta-learning device, the information encoded in the material states of the local environment function to guide active inference by weighting sensory inputs according to their reliability, or salience, and constitute a more general device that allow organisms to learn how to learn, by guiding action–perception cycles (active inference).

### 5.2. An example of niche constructions as meta-learning and ecological inheritance

A good example of simple DNC and situated learning is the process of learning technical skills, for instance the learning of nut cracking skills by young bearded capuchin monkeys (*Sapajus libidinosus*) in Boa-Vista forest (Brazil) [16]. Bearded capuchin monkeys feed on palm nuts, which they must crack using stones as hammers, and other stones or logs as anvils [86]. To learn nut cracking for youngsters is no small business, as it requires all sorts of complex actions, e.g. properly placing the nut on the anvil, maintaining the nut stable throughout the striking action, learning the adequate kind of striking action that keeps the nut in place while also have enough velocity to crack open the nut, etc.

Youngsters spend a great deal of time watching and imitating adults' nut cracking prowess with smaller stones and nut pieces [16]. Adults, in turn, leave material behind them, and, importantly, different sorts of reliable ecological traces like oily residues on the stone anvil site, and pieces of shell, as well as the hammer stones used for nut cracking, which further attract and direct youngsters’ interest to the specifics of the nut cracking site.

These alterations to the environment last for years, even when exposed to inclement climates. Although the learning of the nut cracking technique is partly mediated by attending to adults and imitating them, this, in itself, is not sufficient for the learning. Rather, it is the high fidelity, lasting changes to the niche and the technical artefacts left behind by adults that indirectly biases learning over short and long timescales, thus supporting persistent practice over the course of development [16].

Lasting changes made to the developmental niche enable the acquisition of complex nut cracking skills in youngster Capuchin monkeys, and supports the passing on of traditions, defined as the renewed learning of behavioural patterns by each new generation [36,87]. Crucially, this depends upon the changes to the niche which extend the reach of knowledge transmission through the inheritance of salient information over intergenerational timescales.

In turn, such information transmission depends on two interrelated processes associated to DNC and SNC: (i) the construction, engagement, and re-engagement of the developmental niche, along with the information it comprises; (ii) the slow learning fast inference differential between the environment, and the organism (cf. §4.2.). Engaging the niche leads to the accumulation, e.g. of oily residues and nutshells, which increases the precision of the environment understood as the propensity to influence learning. A precise environment is likely to be passed on to the next generation, and thereby can extend the reach of information transmission.

The sort of (non-genetic) niche inheritance that we describe here is a form of ecological inheritance, which rests on the interplay of two kinds of resources: (i) algorithmic information, and (ii) material and energetic resources [88]. Algorithmic information consists of ‘know-how’-like information encoded by structural and functional features of the niche (typically, genetic information or direct knowledge transmission) [25], which is passed on through standard niche inheritance. Material and energetic resources typically correspond to abiota (e.g. material resources like oily residue). Taken in the context of evolutionary biology (and ecology cf. [89]), algorithmic information is defined as anything that can reduce organisms' uncertainty with regard to possible fitness advantage afforded by the selective environment [90,91]. It eases the exploitation of the environment, and in so doing, can causally influence reproductive success [91]. Crucially, on that view, abiota passed on through ecological inheritance can be a source of algorithmic information, e.g. knowing how to exploit food resources based on the traces left by the activity of members of—current and—previous generations. Following the variational free-energy perspective, we can further define algorithmic information as salient information that secures the optimization (e.g. learning and phenotypic plasticity) of the phenotype throughout development; a meta-learning device that members of previous generations craft through reiterated active inference.

The challenge with algorithmic information is that while its transmission is costly, it is nonetheless a necessary resource [91]. The reproduction of the adaptive life cycle that is secured through development involves myriad environmental manipulations (e.g. building nests, beaver dams and knocking palm nuts), which often require organisms to know-how to engage their environment early in development. Organisms can be informed *a priori*, for instance, through algorithmic information that is bound up with their genetic makeup, but also by having access to external channels of algorithmic information. The inheritance of algorithmic information supposes that previous generations themselves had access to algorithmic information having enabled them, in the first place, to exploit the resources of their environment, so as to be efficient enough to afford passing on this information. The inheritance of algorithmic information is a costly business, as it necessitates of previous generation that they invest metabolic resources for its physical acquisition, storage and use, and transmission [91].

From the point of view of the variational approach, however, algorithmic information, understood as salient information, comes at low cost, as it carries the cognitive burden of precision estimation in development (cf. end of §4.3.), which is one of the main mechanism underwriting organisms’ homeostasis (cf. §3.3). Therefore, because the material and energetic carriers of algorithmic information enable cheaper sensory information processing, algorithmic information can compensate for its cost of transmission. As salient information, it could be modelled as environmental precision, and its role in learning could be explored in simulations similar to the one presented in §4.2. It can be approached quantitatively as information gain cf. §3.3., 4.1.). One could, for instance, test the function of algorithmic information by comparing the changes in variational free energy over time between different organism–niche joint phenotypic spaces, in which agents' differential saliences could vary within and across trials. Variations within trials would represent learning in development (DNC scale), and variation across trials would represent the inheritance of more or less precise algorithmic information (SNC scale).

### 5.3. Critiques revisited and predictions for future research

**Figure 4.**
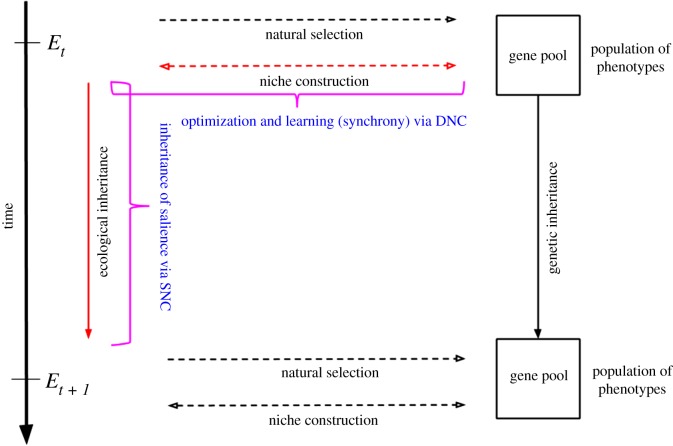
Summary schematic based on [92]. Coloured elements are those that we cover in the paper. The dotted arrows represent the directionality of causal processes. The curly brackets point to the variational processes to which we attribute the content of the bracket. Note that the dotted arrows indicating the directionalities of niche construction points *both ways* (as opposed to the original schematic of [92]). The reason of this is that niche construction, seen through the lens of active inference, entails the reciprocal optimization of the organism(s) and the environment (complementarity) over development (DNC timescale). Therefore, optimization through niche construction at the scale of development is not unidirectional (just like in phylogeny), as the environment *per se* learns and optimizes with respect to the organisms it hosts. Overtime, changes to the environment ‘sediment’, as it were, and become salient information. This information is passed on as algorithmic information through ecological inheritance (SNC timescale) to further guide reciprocal optimization (i.e. phenotypic plasticity and environmental learning).

We conclude our discussion by considering the challenges to NCT presented earlier in light of the variational approach to niche construction (see figure 4 for a visual summary). We examine some of the predictions of the variational approach to niche construction with regard to (i) the generation of non-random, organism-dependent biases on selection pressures; (ii) the consolidation of organism–niche complementarity across temporal scales; and (iii) the cross-generational niche stabilization of environmental conditions.

(i) As a corollary of active inference, niche construction has a systematic causal influence on fitness, on average and over time, as it optimizes the attunement of the organism with its local environment. This is especially important in organisms for which fitness depends on the ability to navigate and attune to complex social environments, and participate in immersive patterned cultural practices, such as humans [37]. Niche construction induces changes to the environment that are not (necessarily) random. On the contrary, many of these changes (although they may be implicit) are continuous with the organism's expectations (the set of priors embodied by the organism), as they result from active inference, which is controlled by these expectations. We can further predict that some of these changes will be deeply continuous with the environmental selection pressures themselves, as these are ‘expected’ by the phenotype of the organism. This is so because natural selection retains sets of priors best adapted to pressures in first place ([20–24] also see electronic supplementary material), which active inference brings forth.
(ii) Active inference allows the organism to specify (often implicitly) those features of the environment that will be adaptive given the demands of their phenotype, i.e. they can specify those features of the niche, the learning of which makes them an accurate model of their environment. On a developmental timescale, this rests on the meta-learning function of niche construction, which guides optimization, or phenotypic plasticity. On the scale of phylogeny, it rests on the inheritance of constraints passed on across generations in the form of species relevant information (salience), which will further guide the optimization in development. Therefore, we can expect the DNC–SNC tandem to tend, over time and on average, to consolidate organism–niche complementarity across timescales.
(iii) The optimization of organism–niche complementarity entails the cross-generational stabilization of the features of the niche, which can be framed as the consolidation of salience: the more agents implicitly engage a material locus of information, the more likely this locus is to further attract engagement, and consequently, the more salience it can acquire. Because of the nature of active inference, organisms tend to learn from (adapt their model to) highly salient environments, potentially deploying culturally patterned practices built around these. Interestingly, we can speculate that an environment that acquires too much salience will become maladaptive, as the attunement dynamics taking place in development require that both the organism and the environment share a certain propensity to learn from one another (c.f. figure 4).

## 6. Concluding remarks

The FEP has motivated the production of much research over the last 10 years. While this work has led to novel theoretical developments, ecologically valid empirical work is forthcoming still. The approach we propose in this paper faces the same limits. So far, it can only be used to make predictions, one of these being that free energy can be cast as an ecological quantity reporting organism–niche complementarity. Much work is still to be done to demonstrate the validity of the variational approach to niche construction. Nonetheless, simulation studies can be used to explore the ecological phase space and to generate predictions about adaptive solutions to ecological problems, framed as free-energy bounding problems. Scientists can proceed on the basis of such predictions and compare these computational models with empirical data. We can further draw from this approach an important conceptual point, which can inform theorizing and empirical work: over time, part of the architecture of priors embodied by members of a population (brain- and body-based priors) becomes encoded in the socio-material setting of the niche. The organism then models an environment which, in turn, models it back, and thereby living systems end up expecting a world that reflects their own expectations. This, however, points to another limit of the variational approach. Thus far, it has only focused on positive feedbacks involved in niche construction, in relation to the adaptation of individual species. Future research should consider the integration of negative feedbacks caused by niche construction, and how those play out in the dynamical relation between natural selection and niche construction, and their consequence in eco-evolution. Moreover, future research should consider the possible limitations of the variational approach with regard to the complexity of ecological inheritance. For instance, modelling the full scope of ecological inheritance might become quickly intractable due to the complexity of the formalism of the variational approach.

## Supplementary Material

Supplementary Material: A Variational Approach to Niche Construction

## Data accessibility

This article has no additional data.
