# Supplementary material for: A variational approach to niche construction
Source: J R Soc Interface. 2018 Apr 11;15(141):20170685. doi: 10.1098/rsif.2017.0685 (PMC5938575; doi:10.1098/rsif.2017.0685)
Supplement: Supplementary Material: A Variational Approach to Niche Construction [file rsif20170685supp1.docx]

**Title:**

Supplementary Material, A Variational Approach to Niche Construction: Natural selection, Bayesian inference, and Bayesian model selection.

**Journal:**

Journal of the Royal Society Interface

**Authors:**

Axel Constant^1,2,3,*^ (email: axel.constant.pruvost@gmail.com)

Maxwell J. D. Ramstead^4,5^ (email: maxwell.ramstead@mail.mcgill.ca)

Samuel P. L. Veissière^5,6^ (email: samuel.veissiere@mcgill.ca)

John O. Campbell^7^ (email: jockocampbell@gmail.com)

Karl J. Friston^8^$\dagger$ (email: k.friston@ucl.ac.uk)

**Affiliations:**

1. Laboratory of Experimental Psychology, Brain & Cognition Unit, KU Leuven, 3000 Leuven, Belgium.

2. Institute of Philosophy, KU Leuven, 3000 Leuven, Belgium.

3. Amsterdam Brain and Cognition center, University of Amsterdam, Science Park 904, 1098 XH Amsterdam, Netherlands.

4. Department of Philosophy, McGill University, 855 Sherbrooke Street West, H3A 2T7, Montreal, QC, Canada.

5. Division of Social and Transcultural Psychiatry, Department of Psychiatry, McGill University, 1033 Pine Avenue, Montreal, QC, Canada.

6. Department of Anthropology, McGill University, 855 Sherbrooke Street West, H3A 2T7, Montreal, QC, Canada.

7. Independent Researcher, Victoria, BC, Canada

8. Wellcome Trust Centre for Neuroimaging, University College London, 12 Queen Square - London, WC1N 3BG, UK.

$\dagger$ Senior author.

**Natural selection, Bayesian inference, and Bayesian model selection**

The aim of this supplementary material is to present the relation between the variational (approximate Bayesian) approaches to model natural selection that we discuss in the text body and well established Bayesian approaches to natural selection.

Central to the variational Free Energy Principle (FEP) is the insight that systems act, in an evidence-driven Bayesian manner, to maximize the evidence supporting their internal models, which describe optimal modes of coupling with the external world. Within the framework of Tinbergen's categories [[function, phylogeny, ontogeny, and mechanism 1]](https://paperpile.com/c/sbEcUb/dU0wG/?prefix=function%2C%20phylogeny%2C%20ontogeny%2C%20and%20mechanism), systems may be optimized in two ways: by evolving more accurate internal models over evolutionary time (phylogeny) or by causing their interactions with the external world to more closely conform to the expectations of their current models (e.g., via active inference in ontogeny, see below) [[cf. 2]](https://paperpile.com/c/sbEcUb/nshwq/?prefix=cf.). Ontogeny instantiates adaptations through mechanisms encoded by the internal model, which function to provide greater fitness or selective advantage. In turn, the evidence of adaptive success or failure updates the existing internal models. Thus, the FEP describes a cyclical causal mechanism where the generative model (genetic or neural) causes self-organized coupling and, in turn, leads to the success of these structures in maintaining the organization of the organism within phenotypic bounds. Thus, Dawkins’ objection [[3]](https://paperpile.com/c/sbEcUb/h8pYb) against natural selection, discussed in the main text, is mitigated; genes may cause phenotypes, but phenotypes also cause genetic changes, as genetic selection takes place the phenotypic level.

Natural selection itself can be cast as a process of variational inference, in the sense that the fittest phenotype for a given ecological niche is also the one most likely to be found in that niche and populate it, given a set of environmental constraints [[2,4–7]](https://paperpile.com/c/sbEcUb/36jGP+r6Cx2+cEVGI+KnE4n+nshwq). Somatic changes can be cast as a Bayesian update of the parameters of the generative model embodied by the organism, and genotypic change through natural selection can be cast as a process of Bayesian model selection [[7]](https://paperpile.com/c/sbEcUb/KnE4n).

The heritable internal models (e.g., sets of priors) of organisms are formed from a number of information processing structures and may take genetic, metabolic, and epigenetic forms [[4]](https://paperpile.com/c/sbEcUb/36jGP). However genetic information appears to provide a top down control algorithm for much of the information processing within the cell [[8]](https://paperpile.com/c/sbEcUb/xALe7). During ontogeny, for example, the execution of this algorithm outputs the phenotype. Thus, we may view genetics in terms of a self-organizing information processing algorithm prescribing the many functions of life. The selection of genotypes can be cast as Bayesian model selection, which determines a model space (phenotype) that can be explored in development (that is, which determines the range of model parameters explored in ontogeny).

All members of a biological species share the same gene sequence, which we might take to be a model of that species. Diploid organisms have two alleles, or versions of the same gene, at each genetic locus and these alleles and their interaction may cause different phenotypic outcomes, for example flower colour. Over evolutionary time, new alleles or even entire new genetic sequences may arise due to errors in copying alleles from one generation to the next. Within a population, a variety of alleles may come to exist at each locus and result in novel phenotypic variations within the species. These variations influence reproductive success, and thus the frequency of a particular allele occupying a given genetic locus may vary over the generations within a population of individuals.

Bayesian descriptions of natural selection are not novel. The change in probability or frequency between generation is due to the relative fitness of the alleles and the process operating to change allele frequencies is natural selection, which is often described mathematically as [[9,10]](https://paperpile.com/c/sbEcUb/kMbNn+ELcp2/?locator=,448):

$q_{i}^{'}= q_{i \frac{w_{i}}{w}}$ (1)

Where $q_{i}^{'}$ is the frequency of the i^th^ allele in the child population, $q_{i}$ is its frequency in the parent population, w_i_ is its fitness and w is the average fitness of all the alleles existing at that locus in the population. This clearly takes the form of a Bayesian update where $q_{i}^{'}$ is the posterior, $q_{i}$ is the prior, and $\frac{w_{i}}{w}$ is the likelihood function; thus natural selection is a process of Bayesian inference [[7]](https://paperpile.com/c/sbEcUb/KnE4n) where the genetic model is updated by evidence regarding fitness provided by the reproductive success of phenotypes.

This Bayesian view on evolution view clearly involves the cyclical causality foregrounded by the variational approach that we espouse. For instance, following Dawkins, and considering beaver dams as a phenotype, genes form an internal model which specifies the design, construction and appropriateness of dams given environmental conditions [[3,11]](https://paperpile.com/c/sbEcUb/AYVyj+h8pYb). Over evolutionary time, variations in the alleles involved explore the design space of possible dams and those hypothetical designs are tested (through ontogeny) and provide evidence of reproductive success when they are output as phenotypes. Genes cause the dams, but reciprocally the reproductive success conferred by dams causes the specific dam-building genes that exist within a current population.

In this view, existing beaver dams, as examples of niche construction, are the result of a thorough exploration of possibilities and their testing, which involves a gradual learning of those designs that confer reproductive success; they are good example of niche construction as adaptation, or extended phenotype [[11]](https://paperpile.com/c/sbEcUb/AYVyj). This is in contrast to mere niche changings, as described by Dawkins, which are often unforeseen consequences of the cyclical causation process considered here. The variational approach to niche construction that we explore in section 3 integrates those seemingly random effects outside of the control of natural selection, under the auspices of the active inference scheme that underwrites free energy minimization.

A description of biology or natural selection in Bayesian terms, from the view of the organisms taking part, must involve very approximate Bayesian procedures, that often use an approximation of the likelihood function as they have little machinery for calculating it directly. Science, on the other hand, from the view of individual scientists, is more direct and can employ estimations with greater accuracy, though, due to the intractability of the likelihood function, many of these solutions (via minimising a free energy bound) are still approximate. For example, experimental design involves maximizing the expected mutual information over possible experimental designs, between the observation and the hypothesis. Such scientific methods that more directly conform to the logic of science (Bayesian inference) may be a primary reason that scientific evolution takes place on a much shorter time scale than does biological evolution.

Rather than rigorous experimental design, biological systems propose new ‘hypothesis’ or ‘models’ (novel genetic structures) for testing at random, within some constraints. Model selection, between competing models, is performed on the basis of relative fitness which is the likelihood function in natural selection [[9]](https://paperpile.com/c/sbEcUb/kMbNn).

The posterior probability of a model M given data D is:

$P\left( M|D \right)= \frac{P\left( D|M \right) P\left( M \right)}{P\left( D \right)}$ , where $P\left( D|M \right)$ is the likelihood function. (2)

Bayes factor gives the ratio between two competing models M_1_ and M_2_:

$K= \frac{P\left( M_{1}|D \right) P\left( M_{2} \right)}{P\left( M_{2}|D \right) P\left( M_{1} \right)}$ (3)

Thus, when models are close to near equal a priori probability the ratio of their likelihood functions (relative fitness) is crucial. In population genetics the prior model probability is the frequency of the genetic model in the existing population, in the parent generation.

The FEP handles this all in its ‘accuracy – complexity’ formulation. See main text. In the Bayesian formulation the ‘complexity’ may be more problematic, but it is tied up with fitness and falls out quite well. As Bretthorst [[12]](https://paperpile.com/c/sbEcUb/smoFa) poses the problem:

“A basic problem in science and engineering is to determine when a model is adequate to explain a set of observations. Is the model complete? Is a new parameter needed? If the model is changed, how? Given several alternatives, which is best? All are examples of the types of questions that scientists and engineers face daily. A principle or theory is needed that allows one to choose rationally. Ockham's razor [1] is the principle typically used. Essentially, Ockham's razor says that objects should not be multiplied needlessly”

We know that novel genetics (model variations) often take the form of gene duplications. This may go to extremes where whole sections of chromosomes may duplicate in a single generation. While these duplications, if they are to persist over time, demonstrate fitness, that fitness comes at the cost of added complexity and provides the potential for greater fitness in the form simplifications which may be achieved through losing some extraneous complexity.

An example is provided with the evolutionary success of flowering plants (angiosperms) over their predecessors the gymnosperms. As argued in [[13]](https://paperpile.com/c/sbEcUb/JN5E), the crucial increase in fitness was achieved through the downsizing of the angiosperm genome. This provided two huge benefits:

1. Smaller cell size allowing a denser concentration of important biological features such as stomata and therefore increased relative fitness.
2. A wider range of complexity available at decreased metabolic costs allowing angiosperms to gain relative fitness through an increased adaptability to specific environmental conditions.

This example may illustrate the manner in which natural selection must balance the advantages of accuracy in its genomic model against the costs of complexity and the manner in which reduced complexity can increases fitness. Jaynes [[14]](https://paperpile.com/c/sbEcUb/hrfyi) observes that a preference for less complex models may also be built into the Bayesian principle of maximum entropy:

“But there is a loose connection between simplicity and plausibility, because the more complicated a set of possible hypotheses, the larger the manifold of conceivable alternatives, and so the smaller must be the prior probability of any particular hypothesis in the set.”

We may conclude that the variational (approximate Bayesian) approaches to model natural selection that we discuss in the text body is generally supported by well established Bayesian approaches to natural selection.

**References**

1. [Tinbergen N. On aims and methods of ethology. Zeitschrift für Tierpsychologie. 1963;20: 410–433.](http://paperpile.com/b/sbEcUb/dU0wG)

2. [Ramstead MJ, Badcock PB, Friston KJ. Answering Schrödinger’s question: A free-energy formulation. Phys Life Rev. 2017; doi:](http://paperpile.com/b/sbEcUb/nshwq)[10.1016/j.plrev.2017.09.001](http://dx.doi.org/10.1016/j.plrev.2017.09.001)

3. [Dawkins R. Extended Phenotype – But Not Too Extended. A Reply to Laland, Turner and Jablonka. Biol Philos. Kluwer Academic Publishers; 2004;19: 377–396. doi:](http://paperpile.com/b/sbEcUb/h8pYb)[10.1023/B:BIPH.0000036180.14904.96](http://dx.doi.org/10.1023/B:BIPH.0000036180.14904.96)

4. [Friston KJ. The free-energy principle: a unified brain theory? Nat Rev Neurosci. 2010;11: 127–138. doi:](http://paperpile.com/b/sbEcUb/36jGP)[10.1038/nrn2787](http://dx.doi.org/10.1038/nrn2787)

5. [Friston KJ, Ao P. Free energy, value, and attractors. Comput Math Methods Med. 2012;2012: 937860. doi:](http://paperpile.com/b/sbEcUb/r6Cx2)[10.1155/2012/937860](http://dx.doi.org/10.1155/2012/937860)

6. [Ao P. Laws in Darwinian evolutionary theory. Phys Life Rev. 2005;2: 117–156. doi:](http://paperpile.com/b/sbEcUb/cEVGI)[10.1016/j.plrev.2005.03.002](http://dx.doi.org/10.1016/j.plrev.2005.03.002)

7. [Campbell JO. Universal Darwinism As a Process of Bayesian Inference. Front Syst Neurosci. 2016;10: 49. doi:](http://paperpile.com/b/sbEcUb/KnE4n)[10.3389/fnsys.2016.00049](http://dx.doi.org/10.3389/fnsys.2016.00049)

8. [Walker SI, Davies PCW. The algorithmic origins of life. J R Soc Interface. 2013;10: 20120869. doi:](http://paperpile.com/b/sbEcUb/xALe7)[10.1098/rsif.2012.0869](http://dx.doi.org/10.1098/rsif.2012.0869)

9. [Frank SA. Natural selection. V. How to read the fundamental equations of evolutionary change in terms of information theory. J Evol Biol. 2012;25: 2377–2396. doi:](http://paperpile.com/b/sbEcUb/kMbNn)[10.1111/jeb.12010](http://dx.doi.org/10.1111/jeb.12010)

10. [Ricklefs RE. Ecology. Concord, Massachusetts: Chiron Press; 1979.](http://paperpile.com/b/sbEcUb/ELcp2)

11. [Dawkins R. The extended phenotype. Oxford: Oxford University Press; 1982.](http://paperpile.com/b/sbEcUb/AYVyj)

12. [Bretthorst LG. An Introduction to Model Selection Using Probability Theory as Logic. Maximum Entropy and Bayesian Methods. Springer, Dordrecht; 1996. pp. 1–42. doi:](http://paperpile.com/b/sbEcUb/smoFa)[10.1007/978-94-015-8729-7_1](http://dx.doi.org/10.1007/978-94-015-8729-7_1)

13. [Simonin KA, Roddy AB. Genome downsizing, physiological novelty, and the global dominance of flowering plants. PLoS Biol. 2018;16: e2003706. doi:](http://paperpile.com/b/sbEcUb/JN5E)[10.1371/journal.pbio.2003706](http://dx.doi.org/10.1371/journal.pbio.2003706)

14. [Jaynes ET. Probability Theory: The Logic of Science. University of Cambridge Press; 2003.](http://paperpile.com/b/sbEcUb/hrfyi)
